# Removal of aqueous Cr(VI) by magnetic biochar derived from bagasse

**DOI:** 10.1038/s41598-020-78142-3

**Published:** 2020-12-08

**Authors:** Meina Liang, Yanmei Ding, Qing Zhang, Dunqiu Wang, Huanhuan Li, Lin Lu

**Affiliations:** 1 School of Environmental Science and Engineering, Guilin University of Technology, Guilin, 541004 People’s Republic of China; 2 Guangxi Key Laboratory of Environmental Pollution Control Theory and Technology, Guilin, 541004 People’s Republic of China

**Keywords:** Environmental sciences, Environmental chemistry

## Abstract

We prepared a novel adsorbent functionalized by bagasse magnetic biochar (BMBC). To study the removal behaviors and mechanisms of Cr(VI) by BMBC, batch adsorption experiments were conducted by modifying variables, such as pH, adsorption time, BMBC dosages, initial Cr concentration, co-existing ions, and ionic strength, and characterizing BMBC before and after Cr(VI) adsorption. BMBC was primarily composed of Fe_2_O_3_ and Fe_3_O_4_ on bagasse boichar with an amorphous structure. The specific surface area of BMBC was 81.94 m^2^ g^−1^, and the pH_pzc_ of BMBC was 6.2. The fabricated BMBC showed high adsorption performance of Cr(VI) in aqueous solution. The maximum Cr(VI) adsorption capacity of BMBC was 29.08 mg g^−1^ at 25 ºC, which was much higher than that of conventional biochar sorbents. The adsorption process followed pseudo-second-order kinetics and could be explained by the involvement of the Langmuir isotherm in monolayer adsorption. The crystalline structure of Fe_3_O_4_ in the BMBC changed slightly during the adsorption process; Fe_3_O_4_ improved the adsorption of Cr(VI) on BMB. The desorption capacity of Cr(VI) was 8.21 mg g^−1^ when 0.2 mol L^−1^ NaOH was used as the desorption solution. After being reused three times, the removal efficiency is still as high as 80.36%.

## Introduction

Chromium (Cr) is a well-known carcinogen that is ubiquitous in the environment. Cr is primarily released into the environment by the tanning, electroplating, printing, and dyeing industries^1^. It continuously accumulates in the body through the food chain^2^ and can cause liver, kidney failure, and even cancer if Cr concentrations in the body are excessive^3^. Cr usually exists in trivalent and hexavalent forms, and Cr(III) can be converted to Cr(VI) and vice versa under certain conditions. Cr can exist in the environment in various forms, and hexavalent chromium [Cr(VI)] is one of the most toxic forms. Therefore, the removal of Cr(VI) in water has received widespread attention^4^.

Current treatment methods of chromium-containing wastewater include adsorption, precipitation, electrolysis, ion exchange, and membrane separation, each of which is accompanied by various advantages and disadvantages^5–8^. With the development of adsorption technology, researchers have become increasingly focused on developing new, low-cost adsorbents for use in adsorption technology^9,10^. Biochar is considered a simple, highly efficient, and cost-effective technology for the removal of Cr(VI) from water^11^. However, separating solids and liquids during the treatment of chromium with biomass carbon is a major challenge that has limited the practical use of biochar for the treatment of contaminated water^8,12^. Magnetization provides a potential solution for overcoming this difficulty given that the products of this process show strong paramagnetic and high saturation properties, which facilitate the separation of solids and liquids^13,14^.

Zhang Xin et al.^15^ prepared tobacco petiole biochar by pyrolysis to remove Cr(VI) and achieved a maximal removal rate of 66.7%. Alex Fabiano Cortez Campos et al.^16^ prepared novel nanoadsorbents based on core–shell bimagnetic nanoparticles that were applied as potential sorbents for Cr(VI) removal and found that the Brunauer–Emmett–Teller (BET) of nanoadsorbents was 152.6 m^2^ g^−1^ and that the maximum adsorption capacity was 15.6 mg g^−1^. Yi Yunqiang et al.,^17^ prepared four types of magnetic biochars for the removal Cr(VI) in steel pickling waste liquor. Most of the adsorbed Cr(VI) was reduced, and the remainder of the Cr(VI) was complexed with C=O groups in magnetic biochar in their studies. Chen Youyuan et al.^18^ used *Enteromorpha prolifera* as a raw and magnetically modified biochar and achieved a maximum removal efficiency of 97.71% for 100 mg L^−1^ of Cr(VI).

In this study, bagasse magnetic biochar (BMBC) was prepared by co-precipitation synthesis with bagasse as the raw material and iron oxide as the modified reagent. The effect of BMBC was studied under different pHs, dosages of BMBC, initial Cr(VI) concentrations, and adsorption times. The competitive influence of representative ions on Cr(VI) adsorption efficiency was studied individually and in synthetic wastewater. Moreover, the regeneration capacity and reusability of BMBC were examined. The ability of BMBC to be recycled through biochar preparation has great potential for pollutant removal and implications for the environment. Lastly, the kinetics, isotherms, potential mechanisms, and Cr(VI) adsorption in the removal process were examined.

## Experimental

### Chemicals and instruments

All chemicals, including K_2_Cr_2_O_7_, H_2_SO_4_ and H_3_PO_4_ were purchased from Sigma-Aldrich. The BMBC powder diffractometer was used to record the X-ray diffractometer (XRD, X’Pert PRO X, Netherlands). The scanning electron microscopy (SEM) images were by Scanning Electron Microscopy (JSM-6380LV, JEOL, Japan). The concentrations of Cr(VI) were determined by Inductively Coupled Plasma Optical Emission Spectrometer (ICP-OES, Perkin Elmer, optima 7000DV, USA). IR was characterized using Fourier-transform infrared spectrophotometry (PerkinElmer, CAT500A, UK), XPS was characterized by X-ray photoelectron spectroscopy (ESCALAB 250Xi Massachusetts, USA), and EDS was characterized by energy-dispersive X-ray spectrometry (IE350, Oxford, England).

### Preparation of BMBC

(1) *Wash and sift*. Bagasse, in which sugar had fully been washed out, was dried in an oven at 85 °C, pulverized using a universal crusher, and screened using a 0.085-mm standard sieve.

(2) *Impregnation with ferric solution*. Ferrous sulfate solution (0.15 mol L^−1^) was added into a reagent bottle with a wide and frosted mouth along with 50 g of bagasse. After mechanical agitation, the sample underwent ultrasonic oscillation for 30 min and was left submerged for 36 h. The ammonia solution (10% v/v) was slowly added into a reagent bottle under mechanical agitation to adjust the pH of the suspended mixture to 8.5. The product was then heated to 85 °C in a microwave oven and filtered to obtain the filter cake. Lastly, the filter cake was washed with ultrapure water until the pH of the washing liquid was approximately 7.0. The filter cake was then placed in a beaker filled with 200 mL of absolute ethanol, oscillated for 30 min, and filtered to obtain the mixture of ferric hydroxide and bagasse.

(3) *Drying and Calcination*. The mixture of ferric hydroxide and bagasse was placed on a porcelain plate and dried at 110 °C to prepare the dry bagasse iron hydroxide mixture. The dry bagasse iron hydroxide mixture was carbonized at 450 °C for 4 h, cooled, ground, and screened using a 100-mm mesh sieve to obtain BMBC.

The preparation of bagasse boichar (BBC) without magnetic iron oxide is described in Supporting Information S1.

### Batch adsorption experiments

First, 0.20 g of BMBC was weighed in a series of 100-mL polyethylene centrifuge tubes, followed by the addition of 50 mL of solution with different Cr(VI) concentrations, which were adjusted to a set pH value with 0.1 mol/L NaOH or HNO_3_. The tubes were then sealed with capsules and placed in a constant temperature water bath oscillator that was shaken for 48 h at a speed of 150 r min^−1^ and a temperature of 25 °C. The solution was filtered after adsorption equilibrium had been reached. A 0.45-μm filter membrane was used to filter the supernatant, and Cr(VI) concentrations were determined by ICP-EOS (PE Optima 7000DV).

The effect of various operating parameters, such as solution pH (2.0, 3.0, 4.0, 5.0, 6.0, 7.0, 8.0, 9.0, 10.0, 11.0, and 12.0), adsorbent dosage (0.001, 0.002, 0.004, 0.006, 0.008, 0.010, and 0.012 g L^−1^), initial Cr(VI) concentration (2, 4, 6, 8, 10, 12, 15, 20, 30, 40, 50, 60, 80, and 100 mg L^−1^), and adsorption time (0.25, 0.5, 0.75, 1, 2, 3, 4, 6, 8, 10, 12, 15, 18, 21, 24, 27, 30, 36, and 48 h), on Cr(VI) adsorption were studied. The effect of coexisting ions, such as NO_3_^−^, SO_4_^2−^, CO_3_^2−^, and PO_4_^3−^, at 10 mg L^−1^ was also studied.

Using the aforementioned experimental procedures, the concentration of Cr(VI) in solution was determined to calculate the adsorption capacity and removal rate:

1 $${\text{Q}}_{{\mathrm{e}}} = \frac{{({\text{C}}_{0} - {\text{C}}_{{\mathrm{e}}} )}}{M}{\text{V}}$$

2 $${\text{Removal}}\,(\% ) = \frac{{C_{0} - C_{e} }}{{C_{0} }} \times 100\%$$

where *Q*_*e*_ (mg g^−1^) is the adsorption capacity; *C*_*0*_ and *C*_*e*_ (mg L^−1^) are the initial and equilibrium Cr(VI) concentrations, respectively; *V* (L) is the solution volume; and *M* (g) is the mass of BMBC.

### Desorption and regeneration experiments

First, 0.20 g of BMBC saturated with Cr(VI) was added to a series of 100-mL plastic centrifuge tubes, along with 50 mL of the regeneration reagent (0.20 M HCl, 0.20 M H_3_PO_4_, 0.20 M NaOH, and 0.20 M NaHCO_3_ solutions). The solution was then placed in a constant temperature water bath oscillator. After shaking for 24 h, the supernatant was filtered using a 0.45-μm filter, and the concentration of Cr(VI) in the filtrate after desorption was measured. Regeneration of the BMBC for reuse was studied from desorption and readsorption experiments in three coherent cycles.

Desorption efficiency (%) and regeneration utilization efficiency (%) were calculated by the following equation:

3 $${\text{Desorption}}\,{\text{efficiency}}\,(\% ) = \frac{{{\text{C}}_{{\mathrm{d}}} {\text{V}}_{{\mathrm{d}}} }}{{({\text{C}}_{0} - {\text{C}}_{{\mathrm{e}}} ){\text{V}}_{{\mathrm{a}}} }} \times 100\%$$

where *C*_*d*_ is concentration of Cr(VI) after desorption, and *V*_*d*_ is the volume of the desorption solution; *C*_*0*_ and *C*_*e*_ (mg L^−1^) are the initial and equilibrium Cr(VI) concentrations, respectively; *V*_*a*_ is the volume of adsorption solution.

## Result and discussion

### Characterization

#### Magnetic analysis

**Figure 1:**
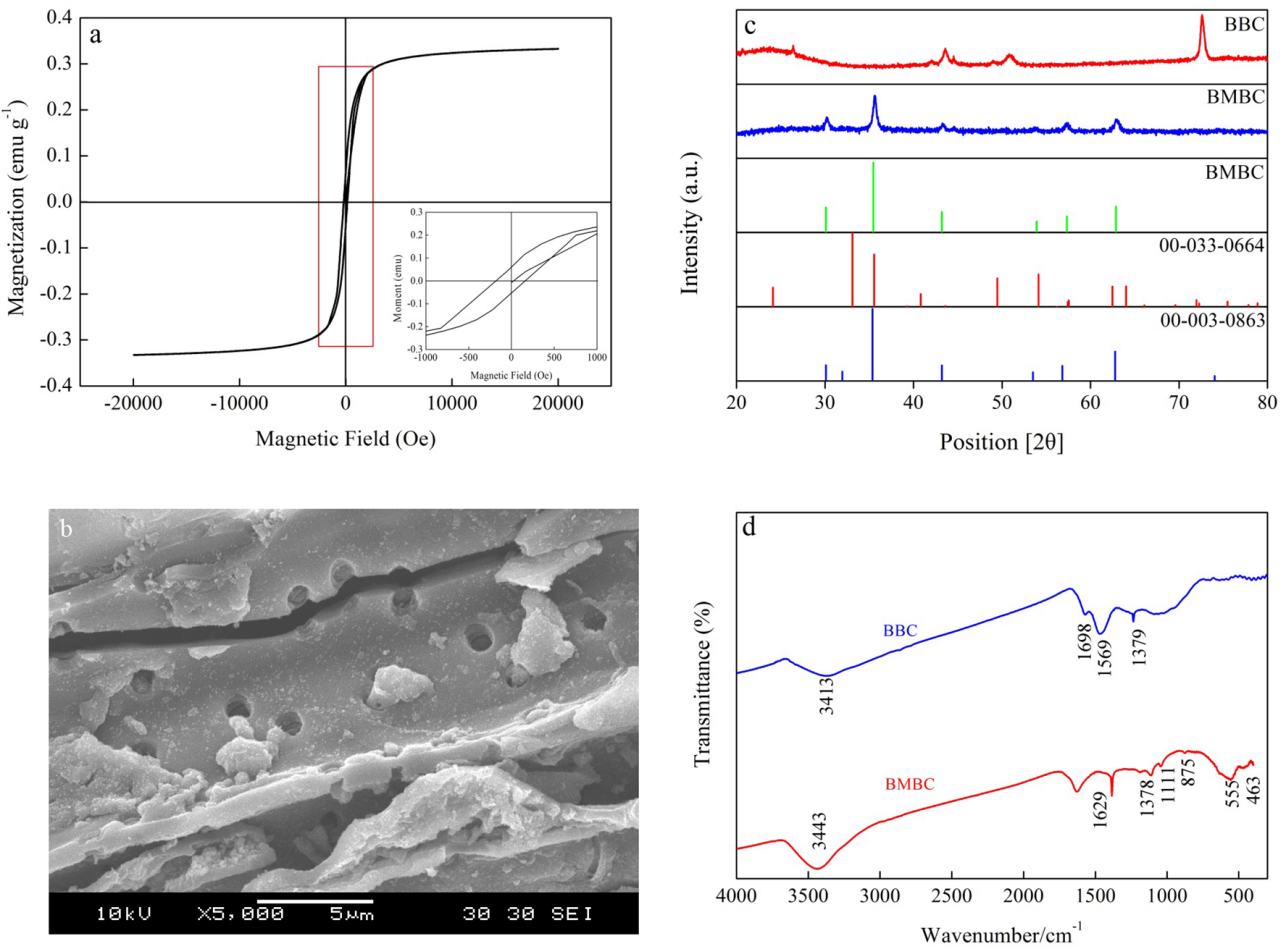
The magnetic hysteresis loops (**a**), SEM (**b**), XRD spectrum (**c**) and FT-IR spectrum (**d**) of BMBC.

The hysteresis loop of BMBC is shown in Fig. 1a. The lower right corner provides a partially enlarged view of the figure. BMBC had magnetic properties, and the hysteresis loops of BMBC were similar to those of nano-magnetic Fe_3_O_4_, indicating that BMBC and Fe_3_O_4_ had the same magnetic properties (Fig. 1a). The saturation magnetization of BMBC was 0.33 emu g^−1^. As the intensity of the applied magnetic field increased, magnetization increased; furthermore, there was a nonlinear relationship between the saturation magnetization and the intensity of the applied magnetic field. When the applied magnetic field was increased, the strength of the applied magnetic field continuously increased, and the magnetization remained constant when saturation had been reached. This pattern can be explained by the fact that the magnetic particles were in a single magnetic domain state. The particles spontaneously magnetized to a saturated state along the easy magnetization direction. Under the action of an external magnetic field, the magnetic moments were neatly aligned. As the applied magnetic field strength increased, the number of particles arranged in the direction of the applied magnetic field increased proportionally with the strength of the applied magnetic field^19^.

### SEM images

SEM images revealed that the surface structure of BMBC was relatively loose; the particle size was uniform, with some particles smaller than 100 nm; and the microporous structure was completely formed (Fig. 1b). The formation of the microporous structure can effectively increase the specific surface area of BMBC.

### XRD analysis

To confirm the existence of iron oxide in the BMBC sample, the BMBC diffraction peak was compared with the Fe_2_O_3_ XRD reference code and the Fe_3_O_4_ reference code of the International Diffraction Data Center (Fig. 1c). The diffraction peaks of BMBC at 2θ = 30.16º, 57.32º, and 62.65º were highly consistent with the diffraction peaks (220), (110), and (214) of the Fe_2_O_3_ reference code (00–033-0664). Furthermore, 2θ = 30.16º, 35.58º, 57.32º, and 62.65º were consistent with the diffraction peaks (220), (311), (511), and (440) of the Fe_3_O_4_ reference code (00–003-0683). At 2θ = 30.16º and 35.58º, there were peaks with larger peak widths and weaker intensities, indicating that smaller Fe_2_O_3_ and Fe_3_O_4_ particles may have been present in BMBC^20^, that the degree of crystallization was low, and that Fe_2_O_3_ and Fe_3_O_4_ were amorphous in structure.

### FTIR analysis

BMBC and BBC corresponded to wavenumbers of 3443, 3413, 1629, 1569, 1378, and 1379 cm^−1^ (Fig. 1d). The wavenumber at 3443 cm^−1^ corresponded to the stretching oscillation of -OH, and the wavenumber at 1629 cm^−1^ was attributed to the characteristic =C=O double bond. The strong peak at 1378 cm^−1^ was assigned to the bending vibration of –COOH and C–C contributing to the D-band peak, indicating that the inner and outer surfaces of BMBC had a large number of oxygen-containing functional groups. Compared BBC and BMBC, at 3414/3443 cm^−1^, the strengthening of the –OH stretching oscillation peak mean increased adsorption capacity; at 1379/1378 cm^−1^, the strengthening of the –COOH bending vibration could provide sufficient adsorption sites. These oxygen-containing functional groups could provide chemical adsorption sites that improve the adsorption capacity of BMBC. The wavenumbers at 555 and 463 cm^−1^ corresponded to the Fe–O bond of Fe_3_O_4_, indicating that the adsorbent contained Fe_3_O_4_^21^.

### BET analysis

The BET specific surface area of BMBC was 81.94 m^2^ g^−1^, and the pore volume was 26.74 cm^3^ g^−1^. The specific surface area of BMBC was approximately 30 times the specific surface area of oak biochar^22^, six times the specific surface area of wheat straw and spruce wood biochar^23^, and approximately 2.4 times the specific surface area of rice husk biochar^24^. By contrast, the BET specific surface area of BBC was 10.19 m^2^ g^−1^, and the pore volume was 3.05 cm^3^ g^−1^, the surface of BMBC had a structure rich in pores, and this finding was consistent with the results of the SEM analysis.

### Zeta analysis

**Figure 2:**
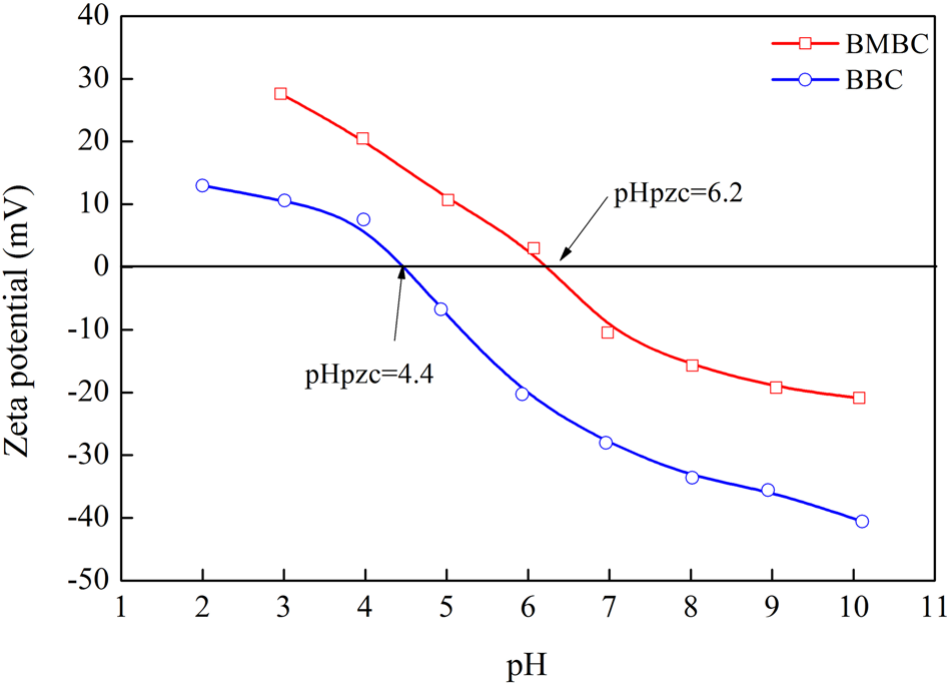
Zeta potential of BMBC as a function of pH.

When the Zeta potential was equal to zero, the point zero charge (pH_pzc_) of BMBC is obtained at the intersection of the pH-Zeta potential curve and the abscissa. Thus, the pH_pzc_ of BMBC was determined to be 6.2, and the pH_pzc_ of BBC was 4.4 (Fig. 2). The pH_pzc_ of BMBC was lower than that of most types of commercial activated carbon^25^ as well as the pH_pzc_ of iron oxides^26^. This difference may have stemmed from the synthesis of BMBC, which was caused by adjusting the pH of the reaction end point with ammonia^27^.

### Error analysis

The kinetic and isotherm parameters were estimated using their respective nonlinear models, which were fitted by Origin 9.0 software (Massachusetts, USA). The fitness confidence was evaluated through several common error analysis methods such as the coefficient of determination (*R*^*2*^)^28^, mean relative error (MRE)^29^,and sum square error (SSE)^30^ [Equations S(1)–S(3)] in the supporting information file.

### Major factors affecting Cr(VI) adsorption

#### Effect of solution pH

**Figure 3:**
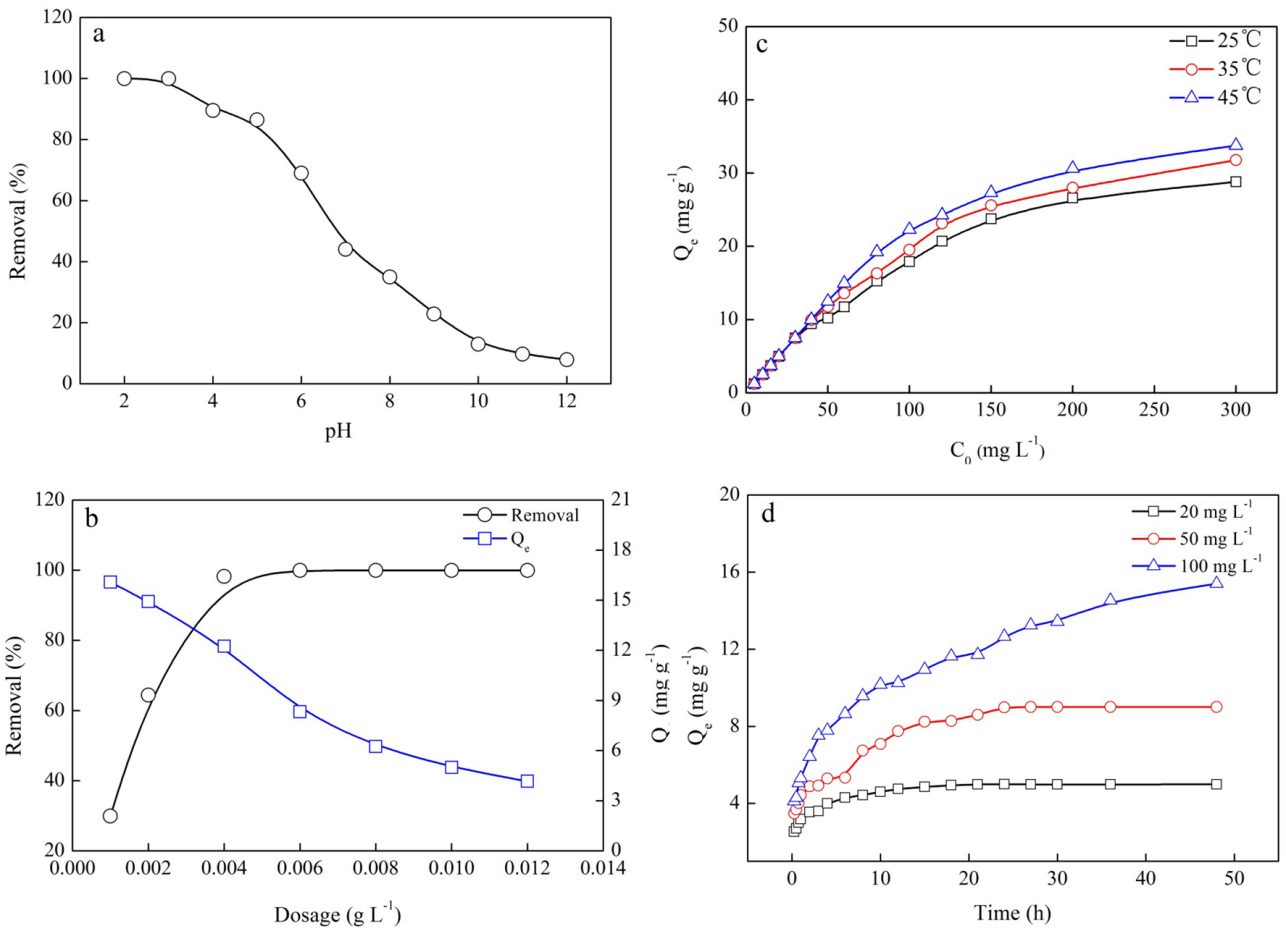
The effect of pH (**a**), dosage (**b**), initial concentration (**c**) and adsorption time (**d**) on the BMBC adsorption of Cr(VI). (**a**) [Concentration of Cr(VI) = 20 mg L^−1^, dosage = 0.004 g L^−1^, solution volume = 50 mL, adsorption time = 48 h, temperature = 25 °C ]. (**b**) [Concentration of Cr(VI) = 50 mg L^−1^, solution volume = 50 mL, pH = 2.0, adsorption time = 48 h, temperature = 25 °C]. (**c**) [Dosage = 0.004 g L^−1^, solution volume = 50 mL, pH = 2.0, adsorption time = 48 h, temperature = 25 °C]. (**d**) [Dosage = 0.004 g L^−1^, solution volume = 50 mL, pH = 2.0, temperature = 25 °C].

The effect of initial aqueous pH on the adsorption of Cr(VI) onto BMBC was studied by varying the pH from 2 to 11 (Fig. 3a). A low pH was beneficial for Cr(VI) removal, and the adsorption capacity of Cr(VI) by BMBC decreased as the pH increased. The removal efficiency decreased from 99.9% to 89.9% as the pH increased from 2.0 to 4.0, respectively. At a pH of 11.0, the removal efficiency decreased to 9.7%. The complete removal of Cr(VI) could be achieved when pH ≤ 4.0.

The pH causes changes in the surface charge of the adsorbent and the degree of protonation of the functional groups on the adsorbent, and the form of Cr(VI) in the adsorption system. When the pH ranged from 2 to 8, the main forms of chromium were Cr^3+^, Cr^2+^, Cr_2_O_7_^2−^, and HCrO_4_^−^^31^. Chromium species were mainly present as CrO_4_^2−^ in water at pH > 8. Dominant chemical reactions in the solution were the following^32^:

4 $${\text{Cr}}_{2} {\text{O}}_{7}^{2 - } + {\text{H}}_{2} {\text{O}} \to 2{\text{HCrO}}_{4}^{ - }$$

5 $${\text{HCrO}}_{4}^{ - } \to {\text{CrO}}_{4}^{2 - } + {\text{H}}^{ + }$$

6 $$2{\text{CrO}}_{4}^{2 - } + 2{\text{H}}^{ + } \to {\text{Cr}}_{2} {\text{O}}_{7}^{2 - } + {\text{H}}_{2} {\text{O}}$$

7 $${\text{Cr}}_{2} {\text{O}}_{7}^{2 - } + 14{\text{H}}^{ + } + 6{\text{e}}^{ - } \to 2{\text{Cr}}^{3 + } + 2{\text{H}}_{2} {\text{O}}$$

8 $${\text{CrO}}_{4}^{2 - } + 4{\text{H}}_{2} {\text{O}} + 3{\text{e}}^{ - } \to {\text{Cr}}\left( {{\text{OH}}} \right)_{3} + 5{\text{OH}}^{ - }$$

At pH < 6.20 (pH_pzc(BMBC)_ = 6.20), positive charges on the surface of BMBC were abundant, as a large number of H^+^ accumulated on the BMBC, making the surface of BMBC highly protonated^31^ and favoring the attraction of Cr(VI). In contrast, when pH was increased to 6.20, the surface of the biochar was negatively charged, and it repelled anionic Cr(VI) in the solution. In addition, the concentration of OH^−^ increased as the pH increased, and it competitively adsorbed Cr_2_O_7_^2−^, HCrO_4_^−^, and CrO_4_^2−^, which resulted in a decrease in the amount of Cr(VI) adsorbed by the biochar. Experiments suggested that the optimal pH was 2, as this pH resulted in the highest removal rate (99.92%) of Cr(VI) by BMBC.

#### Effect of BMBC dosage

The experimental results of the effects of different dosages on the adsorption effect are shown in Fig. 3b. The removal efficiency increased from 29.9 to 99.9% as the dosage of BMBC increased from 0.001 to 0.012 g L^−1^, respectively, with the adsorption capacity decreasing from 14.9 to 4.16 mg g^−1^ (Fig. 3b). Initially, increases in the dosage of BMBC led to increases in the total specific surface area of the adsorbent, resulting in the increased adsorption of Cr(VI) and thus a rapid increase in removal efficiency. At a BMBC dosage of 0.004 g L^−1^, the removal efficiency was 98.20%, and the adsorption capacity was 9.01 mg g^−1^. The removal tended to be stable when the dosage of BMBC exceeded 0.004 g L^−1^, but the adsorption capacity decreased to 4.16 mg g^−1^. Thus, the optimal dosage of BMBC was 0.004 g L^−1^ for wastewater with a Cr(VI) concentration of 50 mg L^−1^.

#### Effect of initial Cr(VI) concentration

The absorption of Cr(VI) in BMBC increased when the initial Cr(VI) concentration increased from 5 to 300 mg L^−1^ at 25, 35, and 45 °C, with the uptake of Cr(VI) increasing from 1.25 to 24.83 mg g^−1^, from 1.25 to 29.78 mg g^−1^, and from 1.25 to 37.27 mg g^−1^, respectively (Fig. 3c). Thus, more Cr(VI) was adsorbed by BMBC as the initial Cr(VI) concentration increased. This pattern may be driven by the fact that when the initial Cr(VI) concentration was increased, the concentration difference between Cr(VI) in the solution and the adsorbent increased, which might have increased the force and thus rapidity of mass transfer between the liquid and solid phases, promoted the migration of Cr(VI) to the surface of BMBC, and thus further improved the adsorption reaction. When the temperature was increased from 25 to 45 °C (Fig. 3c), the adsorption capacity of Cr(VI) by BMBC also increased from 17.94 to 22.33 and from 26.62 to 30.63 at initial Cr(VI) concentrations of 100 mg L^−1^ and 200 mg L^−1^, respectively. These increases might stem from the fact that the reaction temperature reduced the viscosity of the solution and the diffusion resistance of Cr(VI) ions, which further improved the adsorption capacity of Cr(VI) by BMBC.

#### Effect of adsorption time

As the adsorption time increased, the uptake of Cr(VI) by BMBC increased gradually and eventually stabilized (Fig. 3d). For the 20, 50, and 100 mg L^−1^ of initial Cr(VI) concentration at 25 ºC and pH 3, the uptake of Cr(VI) was sorbed within the first 2 h at an average sorption rate of 0.0354, 0.0407, and 0.0535 mg/(g min), respectively (corresponding to 71.00%, 54.26%, and 41.71% of the total amount of Cr(VI) sorbed, respectively). The equilibrium times for the adsorption of Cr(VI) on BMBC at initial Cr(VI) concentrations of 20, 50, and 100 mg L^−1^ were 18, 24, and 48 h, respectively. The equilibrium time lengthened as the initial Cr(VI) concentration increased. In the physical sorption process, most of the sorbate was sorbed within a short adsorption reaction time. However, strong chemical binding of the sorbate with sorbent required a longer adsorption reaction time for adsorption equilibrium to be reached. The uptake of sorbate is relatively rapid during the initial stages and shortly after; however, uptake of sorbate gradually slows as the equilibrium time is approached^33^. The initial rapid uptake of sorbate may stem from the large numbers of available vacant sites on the surface of the adsorbent^34^. After the adsorption reaction reached equilibrium, the equilibrium adsorption capacities of initial concentrations of Cr(VI) of 20, 50, and 100 mg L^−1^ were 5.00, 9.01, and 15.41 mg g^−1^, respectively.

#### Effect of co-existing ions

**Figure 4:**
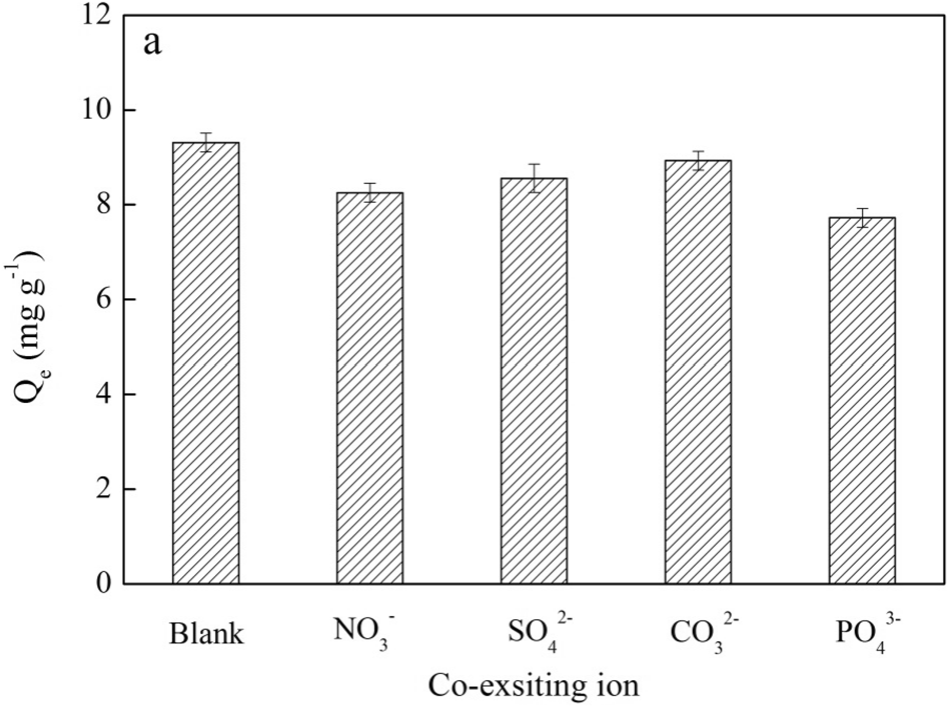
The effect of co-existing anions on Cr(VI) adsorption. [Concentration of Cr(VI) = 20 mg L^−1^, solution volume = 50 mL, pH = 2.0, adsorption time = 48 h, temperature = 25 °C, concentration of NO_3_^−^, CO_3_^2−^, SO_4_^2−^, and PO_4_^3−^ = 10 mg L^−1^].

NO_3_^−^, SO_4_^2−^, CO_3_^2−^ and PO_4_^3−^ at 10 mg L^−1^ in the aqueous solution compete with Cr(VI) for adsorption, reducing the uptake of Cr(VI) by BMBC. NO_3_^−^, SO_4_^2−^, CO_3_^2−^ and PO_4_^3−^ have different effects on the uptake of Cr(VI) (Fig. 4). The degree to which the adsorption capacity of Cr(VI) by BMBC was inhibited by these ions exhibited the following rank order: CO_3_^2−^ > SO_4_^2−^ > NO_3_^−^ > PO_4_^3−^. The negative effect of CO_3_^2−^ on the adsorption capacity of Cr(VI) by BMBC was relatively large. For example, when the concentration of Cr(VI) was 50 mg L^−1^, the adsorption capacity of Cr(VI) decreased from 9.01 to 8.76 mg g^−1^, a decrease of 2.77%. This decrease may stem from the fact that CO_3_^2−^ competed with Cr(VI) for adsorption by BMBC. NO_3_^−^, SO_4_^2−^, CO_3_^2−^ and PO_4_^3−^ have varying degrees of inhibition on the uptake of Cr(VI), which ultimately stem from differences in the affinity between these anions and BMBC^35^.

### Adsorption kinetics

To understand the kinetic mechanism of the adsorption process, pseudo-first-order and pseudo-second-order models were used to fit the kinetic data. The linearized forms of the pseudo-first-order and pseudo-second-order model equations are provided below in Equations S(4) and S(5) in the supporting information, respectively:

Data fitted with pseudo-first-order kinetic equations and pseudo-second-order kinetic equations are shown in Figures S1a and S2b in the supporting information, respectively. All corresponding kinetic parameters of the two models at different initial Cr(VI) concentrations are shown in Table 1. Comparison of the correlation coefficient (*R*^2^) values of the pseudo-first-order equation and pseudo-second-order equation revealed that the *R*^2^ values of the pseudo-second-order equation were closer to 1. At Cr(VI) concentrations of 20, 50, and 100 mg L^−1^, the *R*^2^ values of the pseudo-second-order equation were 0.9995, 0.9940, and 0.9785, respectively. The higher *R*^2^ values of the pseudo-second-order equation implied that the adsorption processes of Cr(VI) on BMBC was controlled by chemical adsorption^36^. In addition, when the initial Cr(VI) concentrations were 20, 50, and 100 mg L^−1^, values of Q_e exp_ were 5.00, 9.10, and 15.41 mg g^−1^, respectively; Q_e_ values of the pseudo-first-order equation were 2.10, 7.96, and 20.58 mg g^−1^, respectively; Q_e_ values of the pseudo-second-order equation were 5.10, 9.47, and 15.30 mg g^−1^, respectively. The Q_e_ values of the pseudo-second-order model were clearly closer to the Q_i exp_ values. Hence, the pseudo-second-order kinetics equation more accurately described the adsorption of Cr(VI) by BMBC, suggesting that chemical adsorption is the main means by which Cr(VI) is adsorbed by the adsorbent.

**Table 1:** The pseudo-first-order and pseudo-second-order equations parameters of BMBC adsorption of Cr(VI).

Parameters	Concentration (mg L^−1^)		
	10	50	100
**Pseudo-first-order equation**			
*K*_*1*_(1/min)	0.0032	0.0034	0.0023
*Q*_*m*_ (mg g^−1^)	2.10	7.96	20.58
MRE (%)	19.33	3.88	11.18
SSE	8.41	1.10	26.73
R^2^	0.9554	0.8707	0.6144
Q_i,exp_	5.00	9.01	15.41
**Pseudo-second-order equation**			
*K*_*2*_	0.0042	0.0008	0.0003
*Q*_*m*_ (mg g^−1^)	5.10	9.47	15.30
MRE (%)	0.67	1.70	0.24
SSE	0.01	0.21	0.01
R^2^	0.9995	0.9940	0.9785

Q_i,exp_ is represent adsorption capacity from experiment.

### Adsorption isotherm

The adsorption isotherms of Cr(VI) by BBC and BMBC at 25, 35, and 45 °C are shown in Figures S2 in the supporting information. The uptake of Cr(VI) was 28.82 mg g^−1^ and increased to 33.78 mg g^−1^ when the temperature increased to 45 °C. These results implied that the adsorption of Cr(VI) by BMBC was an endothermic process^37^. The Langmuir and Freundlich models have been extensively used to study isotherm data. In this study, isotherm data were simulated by the Langmuir and Freundlich models. The Langmuir model assumed that a monomolecular layer was formed when adsorption took place without any interaction between the adsorbed molecules. The linearized Langmuir isotherm model is represented by Equation S(6) in the supporting information. The linearized form of the Freundlich model is expressed by Equation S(7).

The linear plots of the Langmuir and Freundlich models are shown in Figures S3a and S3b in the supporting information, and the parameters (including 1/n and K_L_^38,39^) of both adsorption isotherm models evaluated from the linear plots are shown in Table 2. The adsorption capacity of Cr(VI) was improved after the modification of bagasse biochar. At 25 °C, the maximum adsorption capacity of Cr(VI) by bagasse biochar was 13.15 mg g^−1^, and the adsorption capacity of Cr(VI) by BMBC was 28.82 mg g^−1^, an increase in adsorption capacity of 119.16%. Yu Dezhong^40^ used nanoscale iron oxide to adsorb Cr(VI) and found that the maximum adsorption capacity of Cr(VI) was only 0.3983 mg g^−1^.

**Table 2:** Parameters of Langmuir and Freundlich equations of BMBC adsorption of Cr(VI).

T(°C)	Langmuir equation			Freundlich equation		
	K_L_	R^2^	Q_max_ (mg g^−1^)	K_f_	1/n	R^2^
25	0.1240	0.9757	29.08	9.69	0.182	0.8754
35	0.2062	0.9853	31.37	12.15	0.165	0.8412
45	0.2417	0.9935	33.21	16.33	0.139	0.7242

Langmuir isotherm parameters at 25, 35, and 45 °C showed that the adsorption process partially fitted the Langmuir isotherm model, with correlation coefficients (*R*^2^) of 0.9757, 0.9853, and 0.9935, respectively. This finding demonstrated that monolayer adsorption was the most common way by which Cr(VI) was adsorbed by BMBC. The calculated maximum adsorption, Q_max_, from the Langmuir isotherm model was 29.08, 31.37, and 33.21 mg g^−1^ at 25, 35, and 45 °C, respectively. The correlation coefficients (*R*^2^) of the Freundlich model were 0.8754, 0.8412, and 0.7242 at 25, 35, and 45 ºC, respectively. Comparison of the correlation coefficients between the two adsorption models revealed that the Langmuir adsorption isotherm model better fit the isotherm data for Cr(VI) adsorbed onto BMBC.

The similar adsorbents used for the adsorption of Cr(VI) are summarized in Table 3. BMBC had higher Cr(VI) adsorption capacities (29.08 mg g^−1^) than other previously examined adsorbents (4.61–25.27 mg g^−1^) that have been used to treat Cr(VI) from industrial effluents. Nevertheless, comparisons between adsorbents in their Cr(VI) removal capacities are potentially hampered by differences between the microenvironments of the different solutions^41^.

**Table 3:** The Q_max_ of BMBC evaluated by Langmuir model comparison with other similar adsorbents for Cr(VI) adsorption.

Adsorbent	Q_max_ (mg g^−1^)	pH	Conc. (mg L^−1^)	BET (m^2^ g^−1^)	T (K)	References
MNA-S	15.6	2.5	20–50	152.6	298	^46^
MNA-L	11.1	2.5	20–50	34.4	298	^46^
microalgal based materials	25.19	2	1–10		295	^47^
UV-modified biochar	20.04	Natural pH	20		298	^48^
Melia azedarach wood magnetic biochar	25.27	3.0	5–200	5.219	298	^48^
Oak wood char	4.62	2.0	1–100	1–3	298	^22^
Oak bark char	4.61	2.0	1–100	1.88	298	^22^
Astragalus mongholicus magnetic biochar	23.85	2.0	50	59.34	298	^49^
BMBC	29.08	2.0	20–50	81.94	298	Our study

Changes in the free energy of adsorption were calculated from experiments conducted at different temperatures using Equations S(8) and S(9) (i.e., van’t Hoff equation). Values of the standard enthalpy change (*ΔH°*) and the standard entropy change (*ΔS°*) were determined from the slope and the intercept of the linear plot of ΔG° versus T (Table 4).

**Table 4:** Adsorption thermodynamic parameters.

T (K/°C)	K_L_ (L mg^−1^)	ΔG (kJ mol^−1^)	ΔH (kJ mol^−1^)
298 (25)	0.2594	− 12.1289	35.1039
308 (35)	0.2030	− 12.5741	
318 (45)	0.6366	− 13.1420	

At 25, 35, and 45 °C, the values of ΔG° were − 12.1289, − 12.5741, and − 13.1420 kJ mol^−1^, respectively (Table 4). All values of ΔG° were less than 0, indicating that the adsorption of Cr(VI) by BMBC was spontaneous. The absolute value of ΔG° increased as the temperature increased, indicating that the spontaneous nature of the adsorption process increased as the temperature increased. ΔH° > 0 indicated that the adsorption reaction was endothermic, and increases in temperature were favorable for adsorption.

### Potential adsorption mechanism

**Figure 5:**
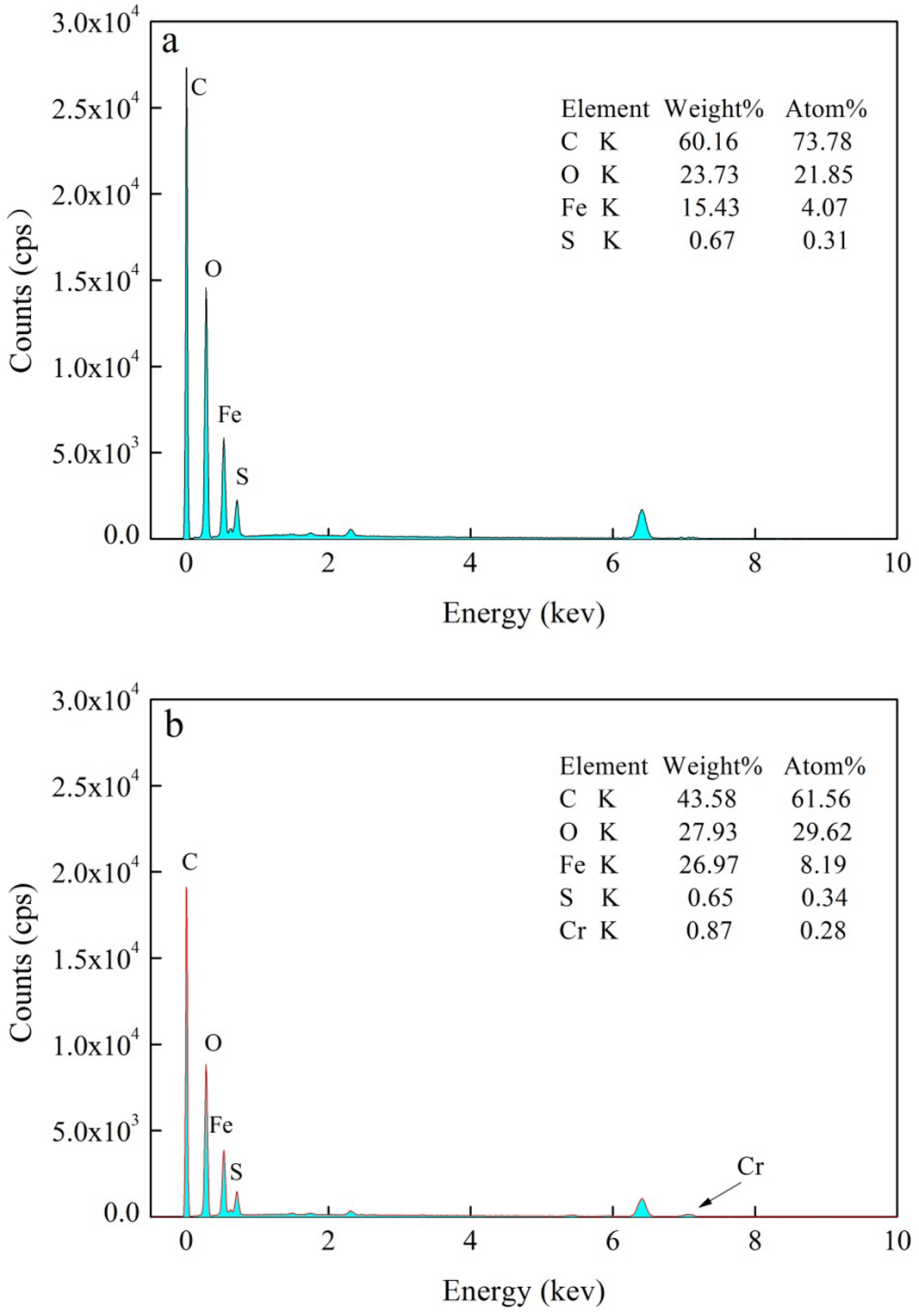
Energy spectrum analysis before adsorption (**a**) and after adsorption (**b**).

The energy spectra of BMBC and BMBC following Cr(VI) adsorption is shown in Fig. 5. The mass percentages (%) of C, O, Fe, and S in the BMBC were 60.16, 23.73, 15.43, and 0.67 (Fig. 5a), respectively; after Cr(VI) adsorption, these percentages were 43.58, 27.93, 26.97, and 0.65 (Fig. 5b). The peak for Cr appeared on the energy spectrum, indicating that Cr existed on the surface of BMBC after Cr(VI) adsorption, and the mass percentage was 0.87%.

**Figure 6:**
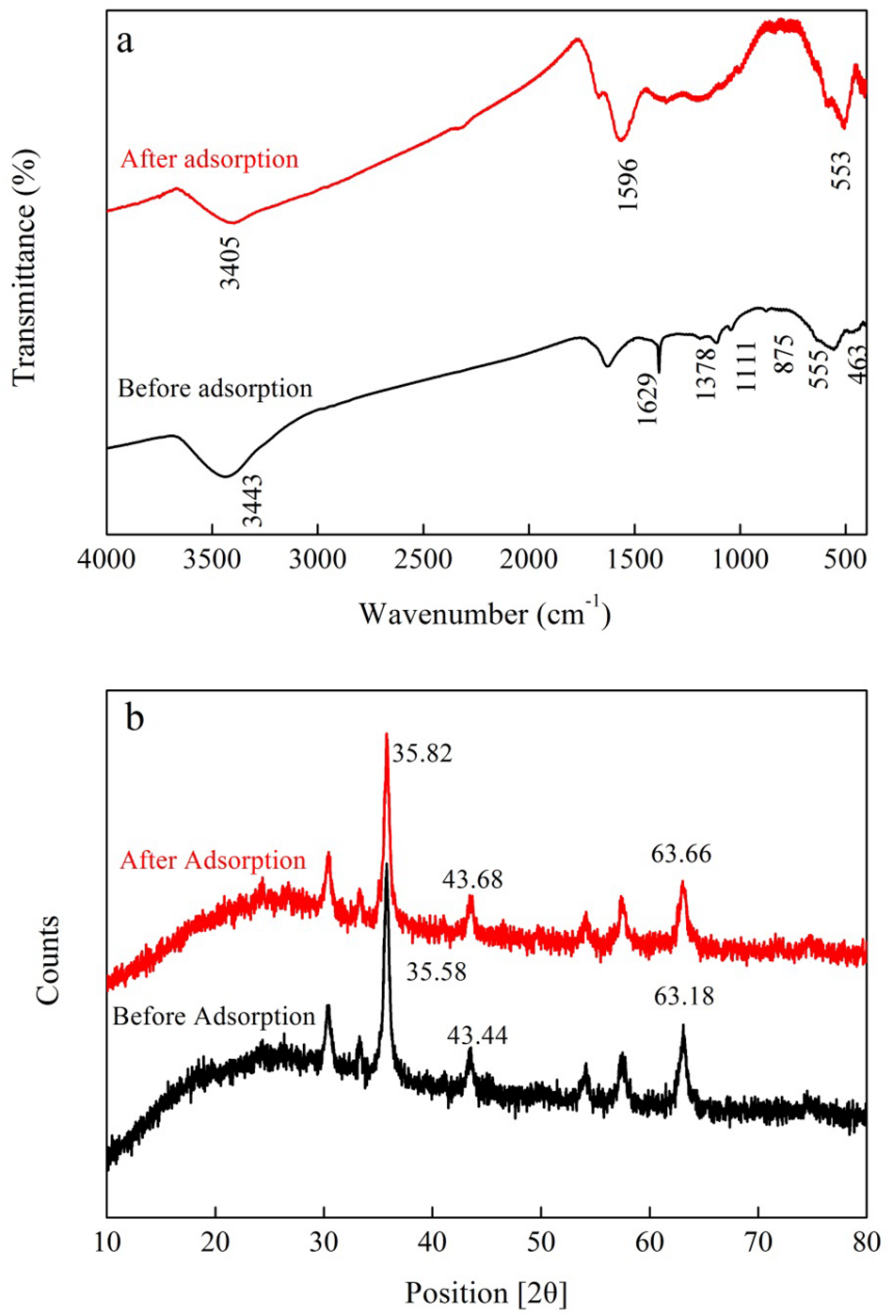
FTIR spectrum (**a**) and XRD spectrum (**b**) before and after the adsorption of Cr(VI) by BMBC.

FTIR analysis was used to characterize Cr(VI)-Cr(VI) interactions (Fig. 6a). After the adsorption of Cr(VI) by BMBC, the peaks for O–H (3443 cm^−1^), C=O (1629 cm^−1^), and C–C (1378 cm^−1^) groups changed slightly, and the O–H (1111 cm^−1^) and C-O (875 cm^−1^) groups disappeared from the FTIR of BMBC after Cr(VI) adsorption, indicating that O–H, C=O, C-O, and C–C were favorable for Cr(VI) removal. The functional groups on the surface of the adsorbent determine its adsorption properties. The content of functional groups on the surface of BMBC significantly increased, improving its adsorption effect.

XRD measurements were also made to study the composition of BMBC before and after Cr(VI) adsorption (Fig. 6b). The characteristic diffraction peaks of BMBC at *2θ* = 35.58°, 43.44°, and 63.18° corresponded to lattice planes of (311), (511), and (440) of Fe_3_O_4_, respectively, After adsorption of Cr(VI), the diffraction peaks of the BMBC at *2θ* = 35.58°, 43.44°, and 63.18° shifted to *2θ* = 35.82°, 43.68°, and 63.66°, respectively. These shifts about diffraction peaks indicated that the crystalline structure of Fe_3_O_4_ in the BMBC had changed slightly during the adsorption process^42^, suggesting that Fe_3_O_4_ contributed to improving the adsorption of Cr(VI) on BMBC.

**Figure 7:**
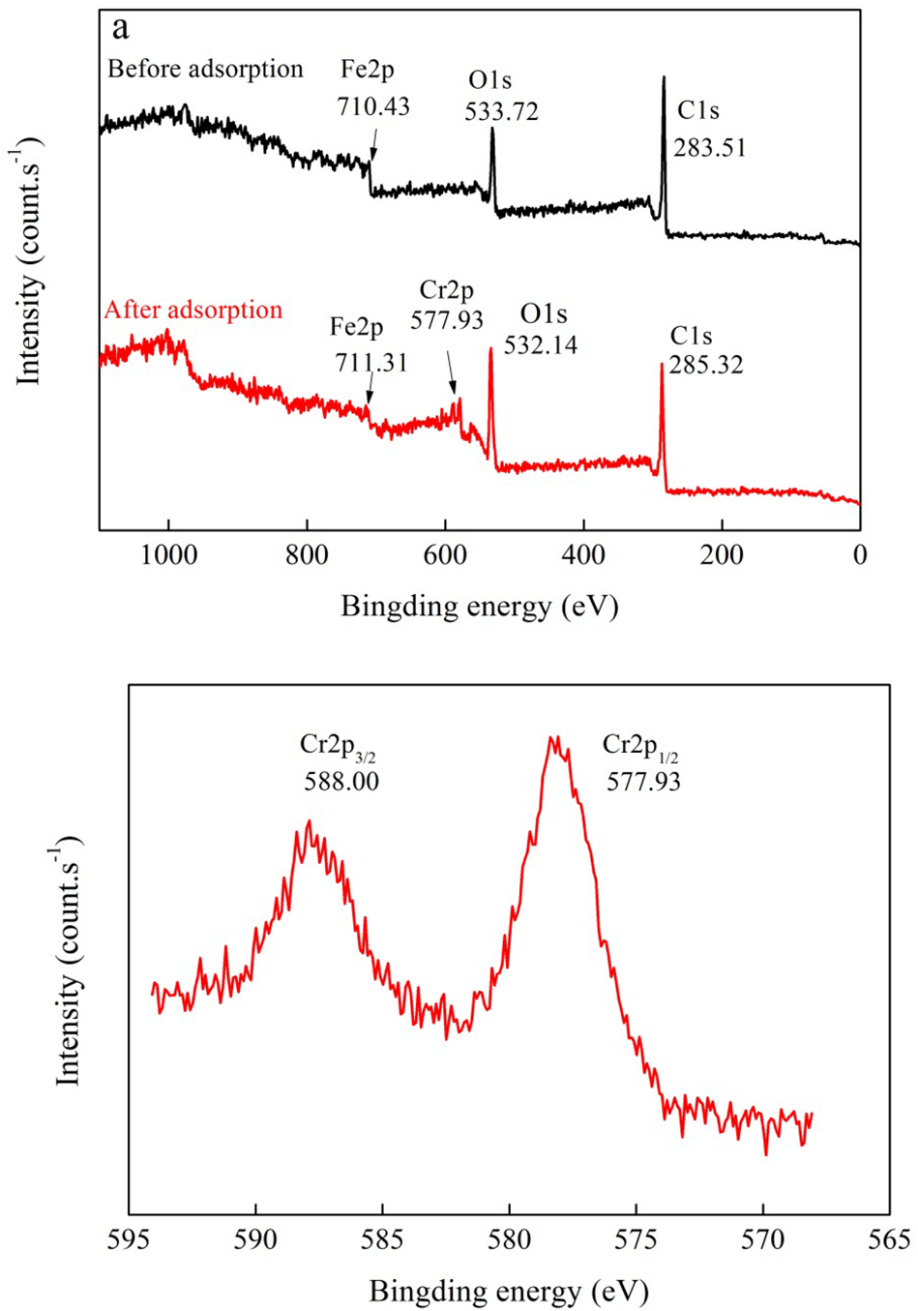
XPS spectra of BMBC before (**a**) and after (**b**) adsorption of Cr(VI).

XPS analysis was used to reveal changes in the valence of Fe, O, C, and Cr before and after Cr(VI) removal (Fig. 7). The adsorbed chromium (Cr 2p) on BMBC was analyzed on the BMBC surface at the atomic level of 3.16% (Table 5). The valences of Cr 2p were separated at 577.93 eV (Cr 2p_1/2_) and 588.0 eV (Cr 2p_3/2_), which was assigned to Cr(III) and Cr(VI), respectively^43^. A part of Cr(VI) was reduced to Cr(III) during the adsorption process; consequently, some Cr(III) was adsorbed onto the surface of BMBC as well. Compared with BMBC, the Fe 2p peaks at 710.43 eV in the spectra of BMBC after Cr(VI) adsorption changed to 711.31 eV, suggesting that Fe 2p participated in the adsorption reaction as dot electrons^33,34^.

**Table 5:** The XPS spectral analysis of before and after adsorption.

Sample	Bond energy (eV)					Composition (%)				
	C1s	O1s	S2p	Fe2p	Cr2p	C1s	O1s	S2p	Fe2p	Cr2p
Before adsorption	283.51	530.72	167.60	710.43	–	73.74	20.62	1.34	4.30	–
After adsorption	285.00	532.14	–	711.31	577.93	66.44	27.93	–	2.48	3.16

### Desorption and regeneration analysis

**Figure 8:**
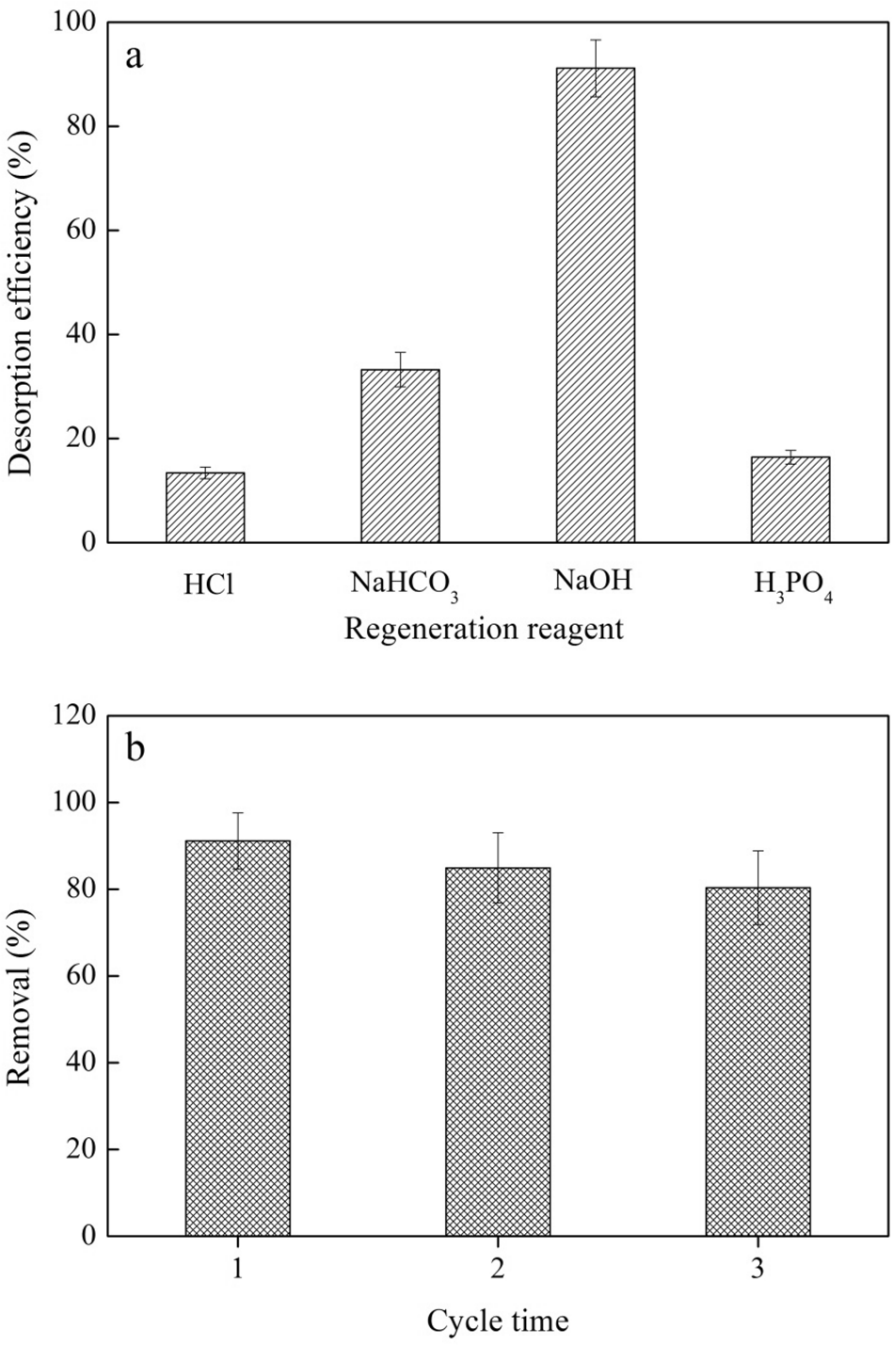
(**a**) Effect of the type of desorption solution on the desorption efficiency and (**b**) regeneration utilization of BMBC with Cr(VI).

The regeneration of BMBC with different reagents (0.2 mol L^−1^ of NaOH, NaHCO_3_, HCl, and H_3_PO_4_) is shown in Fig. 8a. The rank order of the regeneration utilization rate of saturated BMBC after Cr(VI) adsorption was saturated was NaOH > NaHCO_3_ > HCl > H_3_PO_4_. The regeneration utilization rates of the alkaline regeneration reagents (0.2 mol L^−1^ NaOH and NaHCO_3_) were higher than those of the acid regeneration reagents (0.2 mol L^−1^ HCl and H_3_PO_4_). When 0.2 M NaOH was used as the desorption solution, the desorption capacity of Cr(VI) was 8.21 mg g^−1^.

Given the above findings, 0.2 mol L^−1^ NaOH solution was selected for further study as the BMBC recycling regeneration reagent (Fig. 8b). A cyclic adsorption experiment was performed on a Cr(VI) solution with a pH of 2.0 and a concentration of 50 mg L^−1^ to calculate the regeneration utilization rate of the adsorbent. The experimental results are shown in Table 6. After being reused three times, 19.74% of the adsorption capacity of regenerated BMBC was lost, which likely stems from the higher affinity of CrO_4_^2−^ for the BMBC surface compared with OH^−^ ions, further exacerbating the difficulty of ion exchange^44,45^. In practical applications, high-concentration NaOH solutions could be used to increase the OH^−^ concentration and drive the reaction backwards.

**Table 6:** The regeneration recycling experiment of BMBC.

Cycle times	pristine	1	2	3
Adsorption capacity (mg g^−1^)	9.01	8.21	7.65	7.24
Removal (%)	–	91.12	84.91	80.36

## Conclusion

This study examined the removal of Cr(VI) from aqueous solutions by a novel BMBC based on bagasse biochar with magnetic iron oxide. The advantages of the proposed method for BMBC application include its low cost and high reproducibility. The highest percentage of Cr(VI) removal was observed at a pH of 2, with a dosage of 0.20 g of BMBC in a 50-mL solution. The rank order of the degree to which the adsorption capacity of Cr(VI) by BMBC was inhibited was CO_3_^2−^ > SO_4_^2−^ > NO_3_^−^ > PO_4_^3−^. The enhanced adsorption capacity of Cr(VI) on BMBC was explained by the functional groups on the surface of BMBC. The adsorption process followed pseudo-second-order kinetics and could be explained by the involvement of the Langmuir isotherm in monolayer adsorption. The Fe 2p peaks in the spectra of BMBC after Cr(VI) adsorption changed that suggesting that Fe 2p participated in the adsorption reaction as dot electrons. The process was enhanced at higher temperatures, and the adsorption was spontaneous and endothermic. BMBC also showed a high selectivity towards Cr(VI) and a high degree of reusability, which makes it a green material. Based on these results, the BMBC studied in this work demonstrated high efficacy in Cr(VI) removal from water through magnetically assisted chemical separation.

## Supplementary information

Supplementary Information.
