# Supplementary material for: Removal of aqueous Cr(VI) by magnetic biochar derived from bagasse
Source: Sci Rep. 2020 Dec 8;10:21473. doi: 10.1038/s41598-020-78142-3 (PMC7722720; doi:10.1038/s41598-020-78142-3)

**Supporting Information**

**Removal of aqueous Cr(VI) by magnetic biochar derived from bagasse**

Meina Liang ^1, 2^, Yanmei Ding^1^, Qing Zhang ^1, 2*^, Dunqiu Wang^1, 2^，Huanhuan Li^1^, Lin Lu^1^

1. School of Environmental Science and Engineering, Guilin University of Technology, Guilin 541004, P.R. China

2. Guangxi Key Laboratory of Environmental Pollution Control Theory and Technology, Guilin 541004, P.R. China

*Corresponding author: [1291034842@qq.com](mailto:1291034842@qq.com).

Coefficient of determination (*R^2^*), mean relative error (MRE) and sum square error (SSE) as followed:

 S(1)

$MRE(\%)=\frac{100}{n}\sum_{i=1}^{n} \left|\frac{Q_{i,m}-Q_{i,\exp}}{Q_{i,\exp}}\right|$ S(2)

$SSE=\sum_{i=1}^{n} (Q_{i,m}-Q_{i,\exp})^{2}$ S(3)

Note that *Q_i,m_* and *Q_i,exp_* are the calculated and experimental values, respectively.

The linearized forms of the pseudo-first-order and pseudo-second-order model equations are provided below in Equations S(4) and S(5), respectively:

$\ln(Q_{e}-Q_{t})=\ln Q_{e}-\text{K}_{1}t$ S(4)

$\frac{\text{t}}{Q_{t}}=\frac{1}{\text{K}_{2}Q_{e}^{2}}+\frac{t}{Q_{e}}$ S(5)

where *Q_t_* (mg g^-1^) is Cr(VI) uptake at time *t*, and *K_1_* (1/min) and *K_2_* (g mg^-1^ min^-1^) are the pseudo-first-order model and pseudo-second-order model rate constants, respectively.

The linearized Langmuir isotherm model is represented by Equation S(6):

$\frac{\text{C}_{e}}{Q_{e}}=\frac{1}{K_{\text{L}}Q_{m}}+\frac{C_{e}}{Q_{m}}$ S(6)

Where *Q_m_* (mg g^-1^) is the maximum adsorption capacity, *C_e_* (mg L^-1^) is the equilibrium concentration, and *K_L_* is the Langmuir constant related to the energy of adsorption.

The linearized form of the Freundlich model is expressed by Equation S(7):

$\ln Q_{e}=\ln K_{f}+(\frac{1}{n})\ln C_{e}$ S(7)

where *K_f_* is the Freundlich constant related to the energy of adsorption, and the *1/n* constant is related to the intensity of adsorption.

Changes in the free energy of adsorption were calculated from the experiments carried out at different temperatures using Equations S(8) and S(9) (i.e., van’t Hoff equation).

$\Delta\text{G}^{0}=-\text{RT}\ln K_{\text{L}}$ S(8)

$\ln K_{\text{L}}=\frac{\Delta H^{0}}{RT}+\frac{\Delta S}{R}$ S(9)

Where *ΔG°* (kJ mol^-1^) is the Gibbs free energy, *R* is the universal gas constant (8.314 J K^-1^ mol^-1^), *T* (K) is the absolute temperature, *K_L_* corresponds to the thermodynamic equilibrium constant, *ΔH°* (kJ mol^-1^) is standard enthalpy change, and *ΔS°* (kJ mol^-1^ K^-1^) is standard entropy change.

**Figure S1.** The pseudo-first-order (a) and pseudo-second-order (b) equations of BMBC adsorption of Cr(VI)

**
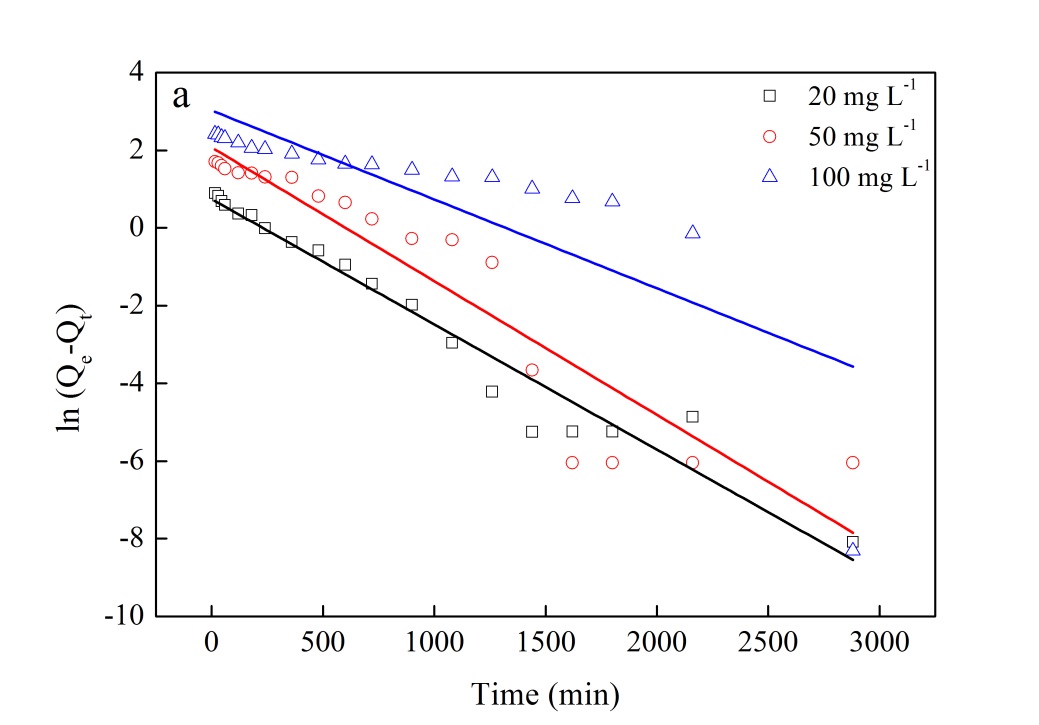
**

**
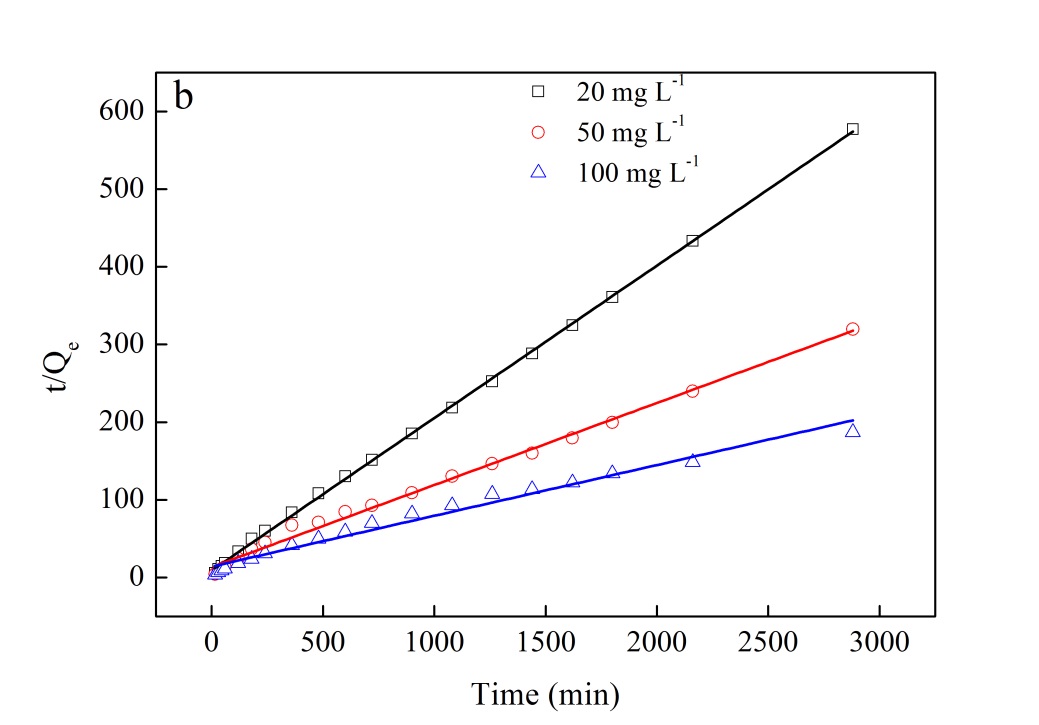
**

**Figure S2.** The adsorption isotherms of Cr (VI) with BBC and BMBC
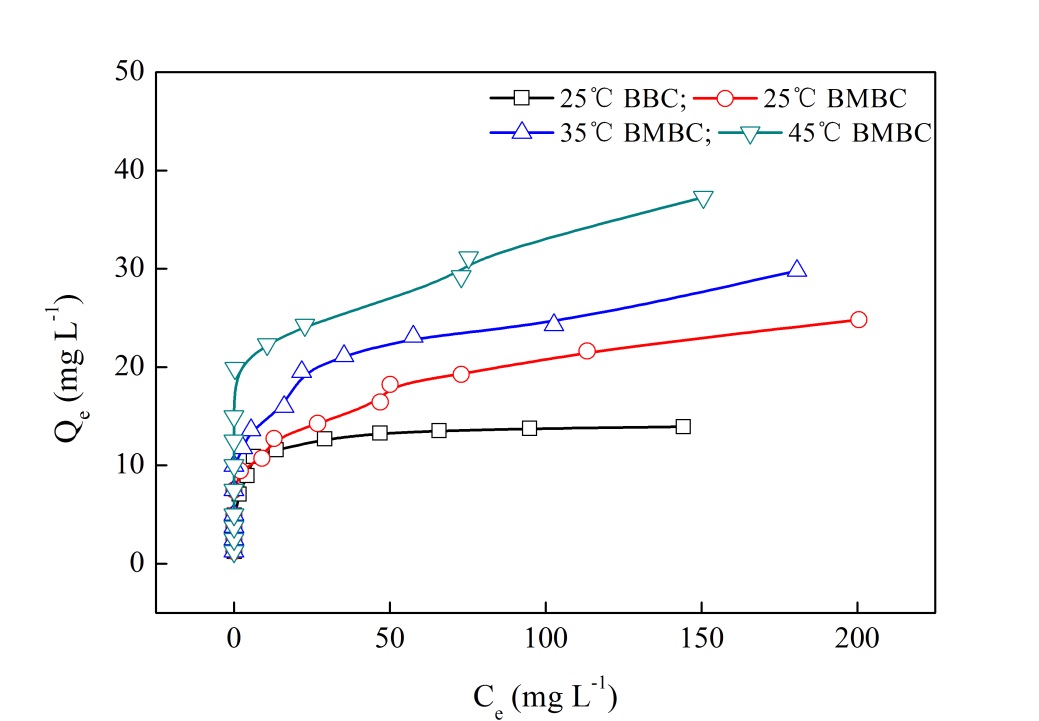


**Figure S3.** Fitting curves of d Langmuir (a) and Freundlich (b) equations of BMBC adsorption of Cr(VI)


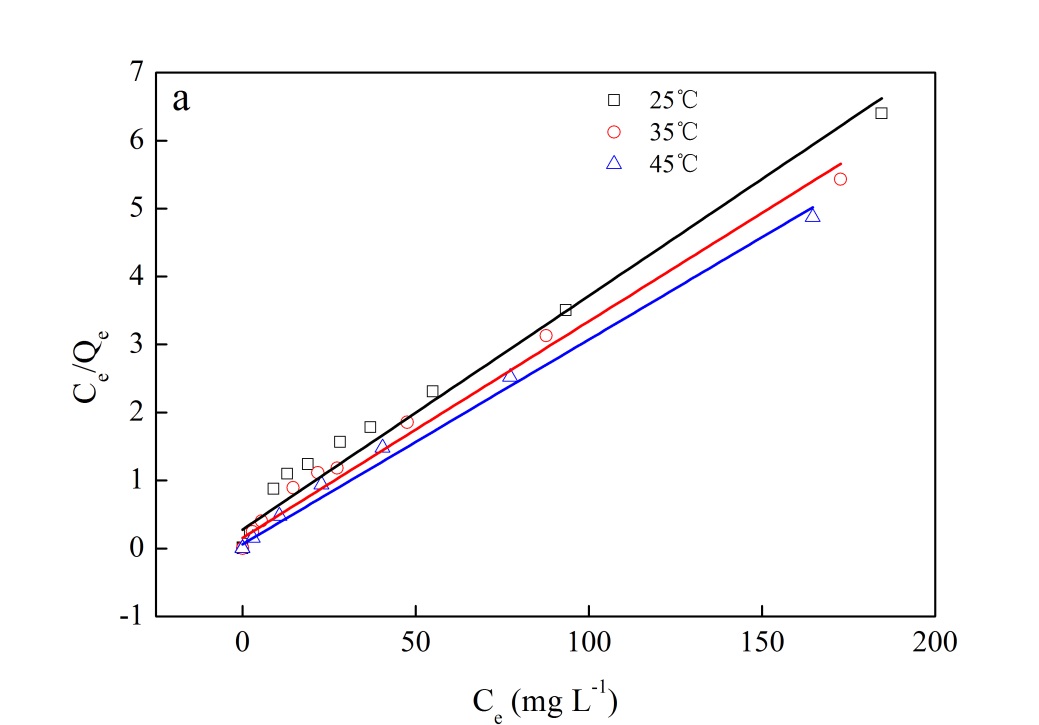


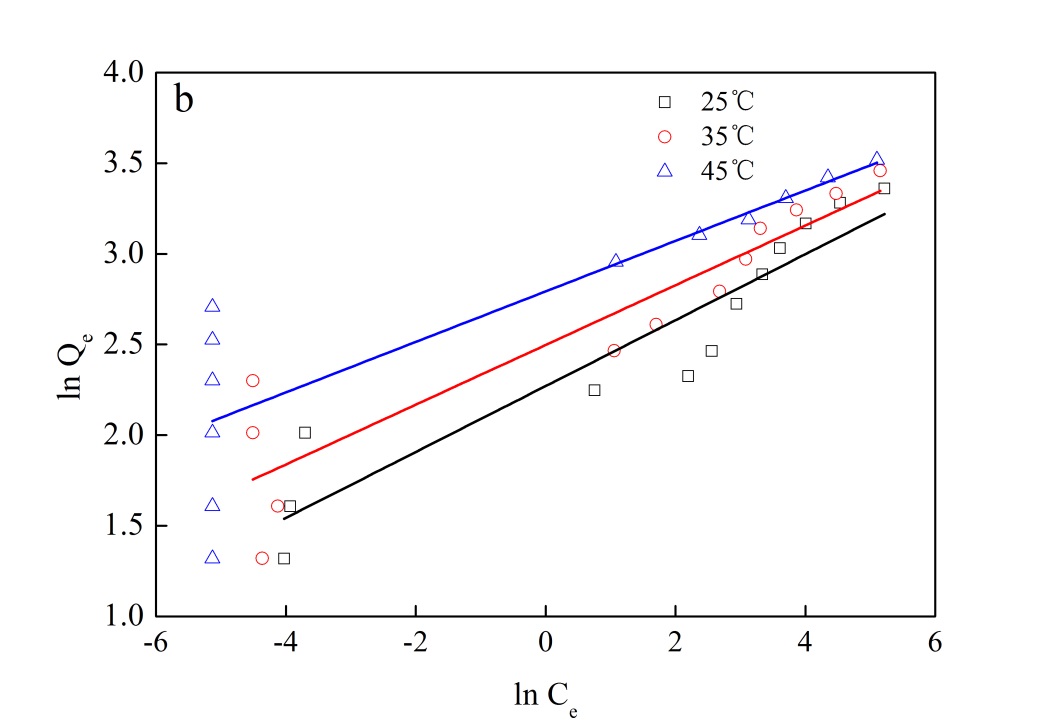

Supplement: Supplementary file 1 — Supplementary Information. [file 41598_2020_78142_MOESM1_ESM.docx]
